# Peptide Sequence Influence on the Conformational Dynamics and DNA binding of the Intrinsically Disordered AT-Hook 3 Peptide

**DOI:** 10.1038/s41598-018-28956-z

**Published:** 2018-07-17

**Authors:** Alyssa Garabedian, Alexander Bolufer, Fenfei Leng, Francisco Fernandez-Lima

**Affiliations:** 1 Department of Chemistry and Biochemistry, Florida International University, Miami, Florida 33199 United States; 2 Biomolecular Sciences Institute, Florida International University, Miami, Florida 33199 United States

## Abstract

The intrinsically disordered ATHP3 was studied at native conditions and in complex with DNA using single amino acid substitutions and high-resolution ion mobility spectrometry coupled to mass spectrometry (trapped IMS-MS). Results showed that ATHP3 can exist in multiple conformations at native conditions (at least 10 conformers were separated), with a variety of proline *cis/trans* orientations, side chain orientations and protonation sites. When in complex with AT rich DNA hairpins, the -RGRP- core is essential for stabilizing the ATHP3: DNA complex. In particular, the arginine in the sixth position plays an important role during binding to AT-rich regions of hairpin DNA, in good agreement with previous NMR and X-ray data. Mobility based correlation matrices are proposed as a way to reveal differences in structural motifs across the peptide mutants based on the conformational space and relative conformer abundance.

## Introduction

The mammalian high mobility group protein (HMGA2, MW ~12 kDa), an intrinsically disordered protein (IDP) that aids in the regulation and expression of certain genes by influencing the remodeling of chromatin structure^1–3^, is also a known oncofetal antigen^1,4^, biomarker of cell transformation and metastasis^5,6^, and a target in cancer therapeutics^7,8^. *In vivo* studies have largely established the physiological role of the full length HMGA2 by monitoring the effects of overexpression and knockouts in mice models^9^. Specifically, HMGA2 gene amplification induces tumorigenesis, as observed in several cancers, including leukemia^10^, tongue cancer^11^, lung cancer^12^, breast cancer^13,14^ and pituitary adenomas^15^. A pygmy phenotype was observed in null mice^6^, whereas the truncated version of the protein is expressed in oversized mice^16^. More recently, *in vitro* analysis implicated HMGA2 in the process of aging^17^. HMGA2 contains three positively charged motifs, known as AT-hook peptides (ATHPs)^18^, that bind with high affinity AT-rich DNA duplex regions^19^, and a negatively charged c-terminal tail. HMGA2 can easily form homodimers^20^, an interaction most likely attributed to contacts forming between the acidic c-terminal tail of one protein with the basic residues of the counterpart protein, and interact with DNA and other proteins, making HMGA2 a protein with a large interaction network^21^. Despite progress made using *in vivo* and *in vitro* studies, there are no structural models for HMGA2, and only a few studies have contributed to the structural characterization (e.g., NMR and crystallography studies) of ATHP bound to duplex DNA^22,23^.

Ion mobility spectrometry and mass spectrometry (IMS-MS) have shown advantages for structural biology, and more specifically, for the study of biomolecules and biomolecular complexes without the influence of the solvent^24–27^. IMS-MS has addressed specific shortcomings of current conventional methods of structural analysis (i.e., NMR and X-ray crystallography) by reducing analysis time, sample consumption, sample purity requirements and restriction on biomolecule flexibility^28,29^. IMS-MS has gained great attention for the study of protein dynamics in their native, partially folded and unfolded forms^24,30–32^, and when complemented with molecular dynamic simulations, has allowed the identification of specific intramolecular interactions that stabilize conformational states (i.e.; proline and side-chain orientation, protonation site)^33^. IMS-MS can capture conformational interconversions and intermediate states for peptides, proteins, DNA and their complexes^24,25,34^. For example, in a recent study, the influence of the solvent composition on the ATHP3 conformational space and ATHP3 kinetically trapped intermediates were reported^33^. At the protein level, IMS-MS studies have shown that phosphorylation of the c-terminal tail can play a vital role during HMGA2 protein-protein and protein-DNA interactions^35^.

In the present study, the peptide sequence influence on the conformational space and DNA binding for the intrinsically disordered ATHP3 is studied using trapped ion mobility spectrometry with collision induced dissociation coupled to mass spectrometry (TIMS-CID-TOF MS) and single amino acid substitutions. A discussion on the conformational space as a function of the protonation sites, side chain and proline cis/trans orientation and the binding affinity to AT-rich DNA follows.

## Methods

### Sample preparation

Native AT-hook peptide 3 (Lys-Arg-Pro-Arg-Gly-Arg-Pro-Arg-Lys-Trp) and all amino acid substituted peptides were purchased from GenScript and used without further purification. An AT-rich DNA oligomer, denoted as FL876, sequence GGATATTGCCCCCGCAATATCC (C_212_H_270_N_79_O_130_P_21_, MW 6655.1561) was purchased from Eurofins Genomics (Luxembourg City, Luxembourg) and used as received. This 22 nucleotide DNA hairpin contains a 9 base pair stem comprised of a 5 base pair AT rich region in the middle of the stem. Solvents and ammonium acetate salts utilized in this study were analytical grade or better and purchased from Fisher Scientific (Pittsburgh, PA). A Tuning Mix calibration standard (G24221A) was obtained from Agilent Technologies (Santa Clara, CA) and used as received.

### Peptide nomenclature

The amino acid sequence of the ATHP3 and variants are presented in Table S1, along with the nomenclature followed throughout the text. Specifically, the variant peptides are referred to by the original amino acid residue followed by their position and the replacement amino acid in a single-code nomenclature.

### Ion Mobility Spectrometry-Mass Spectrometry

Details regarding the trapped IMS (TIMS) operation and specifics compared to traditional IMS can be found elsewhere^33,36–39^. Briefly, a custom nESI-TIMS unit was coupled to a Maxis Impact Q-TOF mass spectrometer (Bruker, Billerica, MA). The TIMS unit is run by custom software in LabView (National Instruments) synchronized with the MS platform controls. Sample aliquots (10 μL) were loaded in a pulled-tip capillary biased at 700–1200 V to the MS inlet. The nitrogen bath gas flow is defined by the pressure differential between the entrance funnel (*P*_1_ = 2.6 mbar) and the exit funnel (*P*_2_ = 1.1 mbar) at *ca*. 294 K. A 880 kHz and 200 V_pp_ rf was applied. Deflector, capillary, entrance funnel, entrance and exit analyzer voltages were 60/−150, 50, 0, −200-0, and 60 V in positive mode (and −60/150, −50, 200-0, and −60 V in negative mode) to prevent ion heating prior to IMS separation. The reduced mobility, K, of an ion in a TIMS cell is described by:

1 $$K=\frac{{V}_{g}}{E}\approx \frac{A}{({V}_{elution}-{V}_{out})}$$

where *v*_*g*_, *E*, *V*_*elution*_ and *V*_*out*_ are the gas velocity, applied electric field, elution voltage and exit analyzer voltage, respectively. After thermalization, species elute from the TIMS cell by decreasing the electric field in stepwise decrements (referred to as the “ramp”) and can be described by a characteristic elution voltage (*V*_*elution*_). The mobility calibration constant *A* was determined using known reduced mobilities of Tuning Mix components (*K*_0_ of 1.013, 0.835, and 0.740 cm^2^/(V.s) for respective *m/z* 622, 922, and 1222). The scan rate (*Sr* = Δ*V*_*ramp*_/*t*_*ramp*_) was optimized for every experiment.

The measured mobilities were converted into CCS (Ω, Å²) using the Mason-Schamp equation:

2 $${\Omega }=\frac{{(18\pi )}^{1/2}}{16}\frac{z}{{({k}_{B}T)}^{1/2}}\,[\frac{1}{{m}_{i}}+{\frac{1}{{m}_{b}}]}^{1/2}\frac{1}{{K}_{0}}\frac{1}{{N}^{\ast }}$$

where z is the charge of the ion, k_B_ is the Boltzmann constant, N^*^ is the number density of the bath gas and *m*_*i*_ and *m*_*b*_ refer to the masses of the ion and bath gas, respectively. TIMS-MS spectra were analyzed using Compass Data Analysis 5.0 (Bruker Daltonik GmbH) and TIMS Data Viewer 1.4.0.31397 (Bruker Daltonics Inc, Billerica). Heat maps of the corrected and normalized IMS profiles were built to facilitate the comparison between the experimental conditions explored.

The IMS corrected profiles were compared using the correlation coefficient function:

3 $$Correl\,(X,Y)=\frac{{\sum }^{}(x-\bar{x})(y-\bar{y})}{\sqrt{{\sum }^{}{(x-\bar{x})}^{2}{(y-\bar{y})}^{2}}}$$

where $\bar{x}$ and $\bar{y}$ are the sample means average for IMS profile 1 and 2. Notice that in this approach, the entire profile is considered to obtain the correlation coefficient.

### Correction of amino acid substituted collision cross sections

For direct comparison of the mobility profiles between the ATHP 3 and single amino acid substituted peptides (e.g., Arg to Ala, Pro to Ala and Trp to Ala), the CCS profiles were adjusted based on the method previously described in references^26,40–42^. To account for differences in CCS between N_2_ and He, the CCS_N2_ = 1.0857 (CCS_He_) + 81.459 [Å^2^] conversion was used^43,44^. This resulted in Arg to Ala, Pro to Ala and Trp to Ala substitutions to be corrected by 17.43 Å^2^, 2.68 Å^2^ and 10.50 Å^2^ in N_2_, respectively.

### Determination of binding affinities

Peptide-DNA binding affinities (K_a_) were calculated using the general equation for an association reaction: peak area of the complex divided by the peak area of the unbound DNA and unbound peptide^45^:

4 $${{\rm{K}}}_{{\rm{a}}}=\frac{\,(Complex)}{(DNA)(Peptide)}$$

A distance matrix was utilized to better evaluate relative changes associated with the single amino acid substitution using the equation:

5 $${\rm{D}}({\rm{X}},{\rm{Y}})=[\frac{{K}_{a(peptid{e}_{X})}-{K}_{a(peptid{e}_{Y})}}{ < \,{K}_{{a}_{peptide(1-8)}} > }]$$

## Discussion

**Figure 1:**
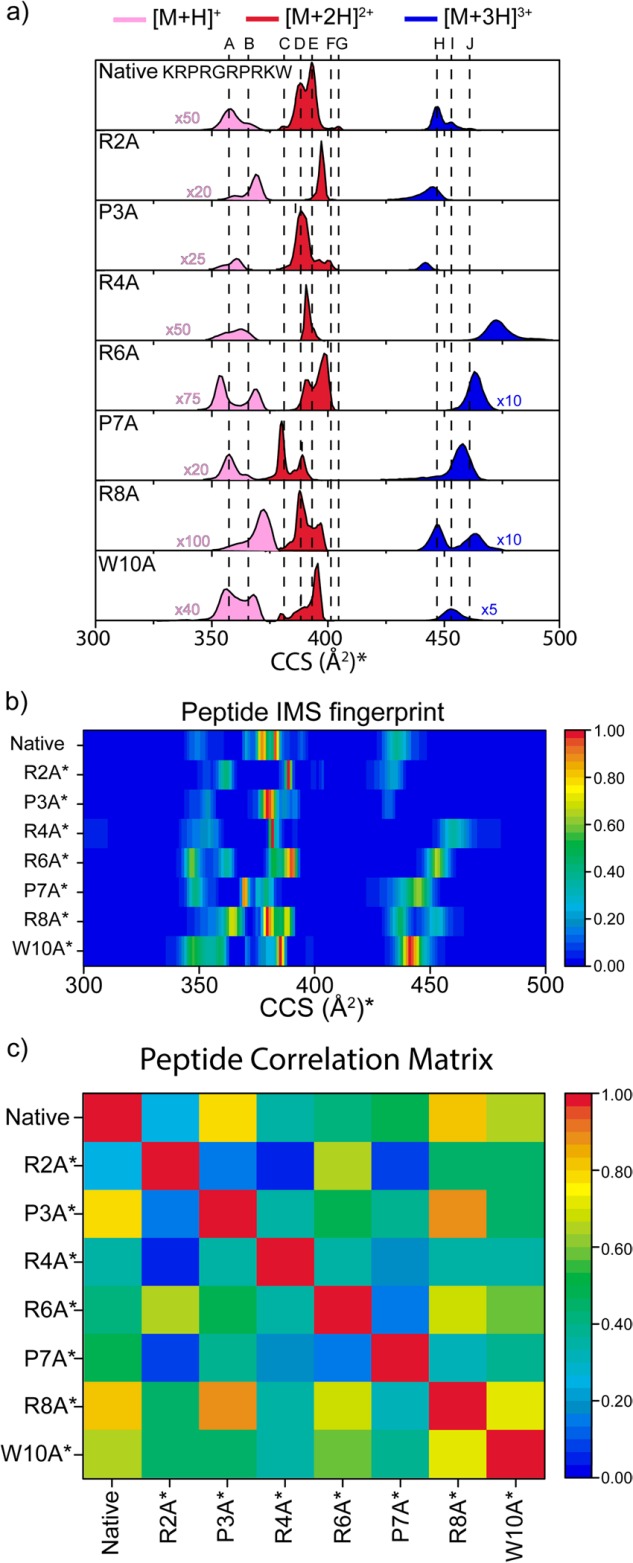
Typical, normalized ATHP3 and corrected variant (*) mobility profiles for the [M + H]^+^, [M + 2 H]^2+^ and [M + 3 H]^3+^ for intrinsic size parameters upon residue substitution to alanine (**a**). Peptide IMS fingerprint (**b**) and correlation matrix (**c**) are used for assessment of the amino acid sequence effect on the secondary structure.

Three charge states in positive ion mode ([M + H]^+^ to [M + 3 H]^3+^) were observed for ATHP3 and the seven amino acid substituted peptides (see corrected CCS profiles and fingerprint in Fig. 1a,b) under native conditions (e.g., 10 mM ammonium acetate, pH ~6.8). A correlation matrix of the IMS fingerprints (Fig. 1c) is used for rapid comparison and identification of the single amino acid substitution that possess the smallest/largest influence in the ATHP3 mobility profile (first column and first row), as well as between the mutants. For example, following the color scale, P3A and R8A are the most and R2A is the least closely related mutants to ATHP3 under native conditions. That is, a quick assessment of the influence of peptide sequence (e.g., single amino acid substitution) on the peptide secondary structure can be perform.

One of the advantages of using single point residue mutations in combination with IMS-MS is the possibility to identify the conformational state of the prolines (i.e., *cis/trans* orientation), the side chain orientations and charge (proton) location by comparison of the corrected IMS profiles^26^. Prolines in the *trans* position require to overcome a ~13 kcal mol^−1^ energy barrier in order to convert to the *cis* position (the *trans* state is ~0.5 kcal mol^−1^ lower in energy than the *cis* form)^46^. However, alanine residues (not bound to proline) are energetically more favorable in the *trans* position (the *trans* state is ~2.5 kcal mol^−1^ lower in energy with a *trans* to *cis* ~20 kcal mol^−1^ barrier^46^). This means that the determination of the ATHP3 *trans* or *cis* state can be accomplished by looking at the conservation (proline in *trans*) or absence (proline in *cis*) of each of IMS band of the native ATHP3 peptide^26^. That is, comparison of the corrected profiles for native, P3A and P7A permits the assignment of the proline bands in the native ATHP3 (results summarized in Table 1). Analogous, we can suggest that a single point mutation of a basic residue (e.g., R) allows for the assignment of the proton location (or charge) in the native ATHP3, since the loss of a charged basic residue (R to A mutation) very likely promotes the disruption of proton-assisted, intramolecular interactions that stabilizes a conformational state. Specifically, the determination of the ATHP3 charge location can be accomplished by looking at the conservation (N) or absence (+1) of the IMS band after the mutation of a basic residue with a neutral residue (e.g., R to A mutation). If an IMS band is present in the ATHP3 and the ATHP3 RxA modified peptides profiles, there is likely no proton-assisted intramolecular interaction at that position. In the case of the ATHP3, up to seven protons can be attached; however, during native conditions (pH ~ 6.7), a maximum of three protons were observed (i.e., +1– +3 charge states). Comparison of the ATHP3 profile with those of R2A, R4A, R6A and R8A permitted the evaluation of intramolecular interactions involving a proton in the basic residue of the native, intrinsically disordered ATHP3 (see Table 1). Knowing the total peptide charge and likelihood of the basic residues to be protonated, the protonation probability of the N-term and the lysine at position one was estimated (i.e., difference from the total charge state and number of protonated arginines). In addition to this information, the W10A substitution provides insight in the role of the tryptophan on the overall conformational space (i.e., is it coordinating or non-coordinating with other residues). Overall, the high number of IMS bands can be attributed to conformations with different locations of the protons (i.e., protomers), potential constraints generated from the proline configuration and orientations of the side chains.

**Table 1:** Summary of charge location, proline cis/trans configuration and side chain orientation (W10) of the Native ATHP3 per IMS band (A–J).

	[M + H]^+^		[M + 2 H]^2+^					[M + 3 H]^3+^		
	A	B	C	D	E	F	G	H	I	J
**Residue**										
R2	+1*	N	+1*	+1*	+1	+1*	+1*	N	+1*	+1
P3	trans	cis	trans	trans	trans	trans	cis	cis	cis	cis
R4	N	N	+1*	+1*	+1	+1*	+1*	+1	+1*	+1
R6	N	N	+1*	+1*	N	N	+1*	+1	+1*	N
P7	trans	trans	trans	trans	cis	cis	cis	cis	trans	trans
R8	+1*	N	N	N	N	+1*	+1*	N	+1*	N
W10	NC	NC	NC	NC	NC	C	C	C	NC	NC
N-ter/K	N	+1	N	N	N	N	N	+1	N	+1

N = neutral. C = coordinated bond. NC = non-coordinated bond. *Denotes cases where charge assignment between basic residues cannot be distinguished.

Inspection of Table 1 shows that ATHP 3 [M + H]^+^ conformers (i.e., A and B bands) differ in the orientation of P3 (*trans* vs. *cis*) and the location of the charge (R2/R8 vs. N-term/K). In the case of ATHP 3 [M + 2 H]^+2^, differences between the five bands (C-G) are also associated to the orientation of the prolines P3 and P7, the protonation of the basic residues R2, R4, R6 and R8, and the coordination of W10. In the case of ATHP [M + 3 H]^+3^, differences between the three bands (H–J) are associated to the proline P7, the protonation of the basic residues R2, R4, R6 and R8, and the coordination of W10. While in some cases the charge residue that is protonated can be uniquely assigned (bands B, E and J), for the other bands we cannot discriminate (denoted with a * in Table 1); that is, different protonation patterns can share the same IMS band. In summary, the data presented in Table 1 shows that the native, intrinsically disordered ATHP3 can exist in a variety of conformations (at least 10), with different proline configurations, protonation sites and side chain orientations.

**Figure 2:**
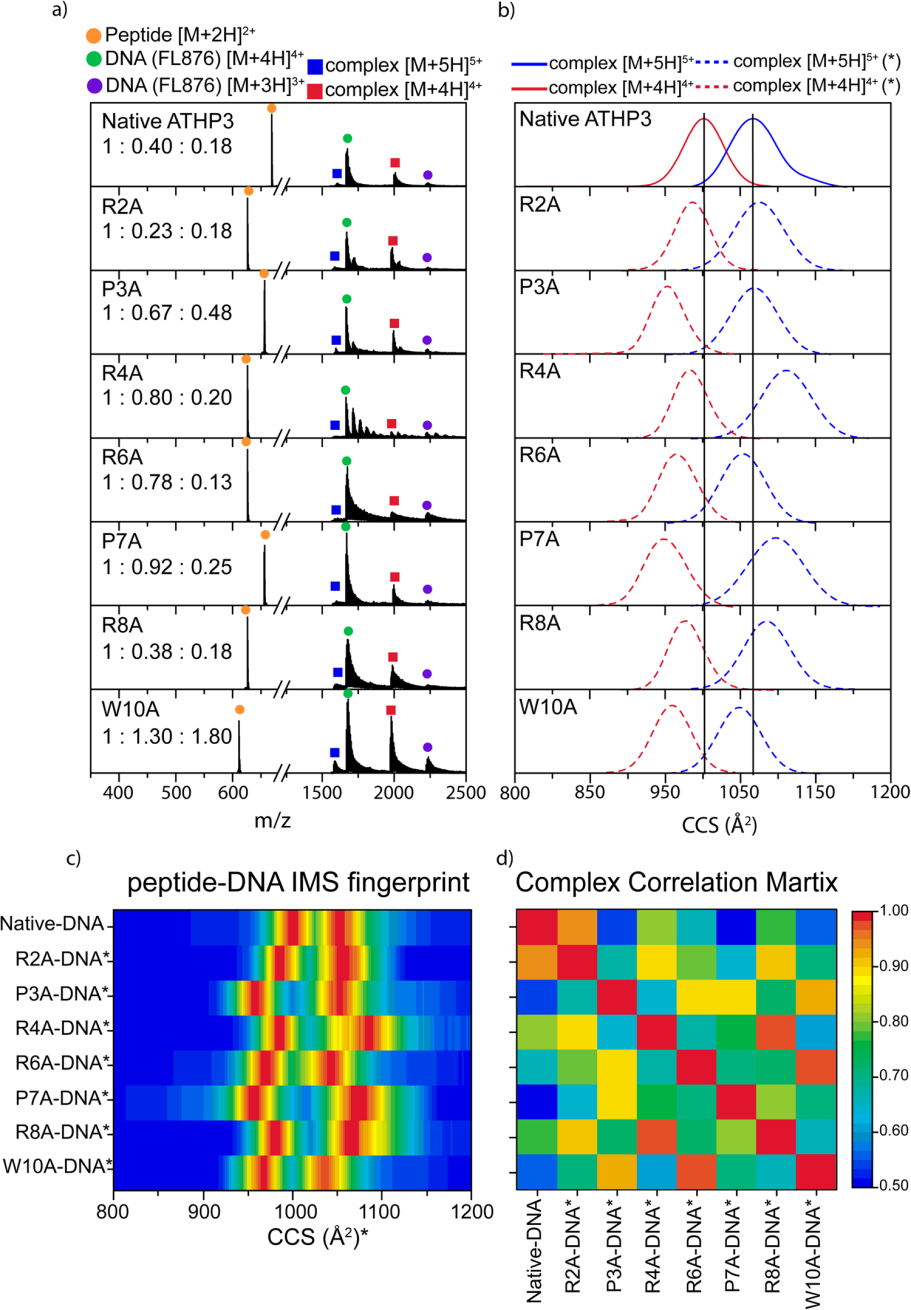
Typical mass spectra (**a**) and ‘corrected’ IMS profiles (**b**) of the native and substituted ATHP3**:** DNA complexes. The IMS profile fingerprint (**c**) of the complexes was used to generate a correlation matrix (**d**). Peptide**:** DNA**:** complex ratios are reported in the inset in (**a**).

Peptide-DNA complexes were formed in solution and observed in the gas-phase at [M + 4 H]^4+^ and [M + 5 H]^5+^ charge states under native conditions for all considered peptide mutants (Fig. 2a). Beside the peptide-DNA complexes, unbound DNA and peptides were also observed at [M + 3 H]^3+^ – [M + 4 H]^4+^ and at [M + H]^+^ – [M + 3 H]^3+^ charge states, respectively. IMS profiles of the peptide-DNA complexes (Fig. 2b,c) can be characterized by a single, broad mobility band. Inspection of the peptide-DNA plots, generated from the corrected IMS profiles, allows for a quick description of the influence of the single amino acid substitution on the structure of the peptide-DNA complex (Fig. 2c). Further information can be extracted from the correlation matrix (Fig. 2d). If the single amino acid substitution does not dramatically affect the corrected IMS profiles (similar fingerprint) or has a high correlation value, then most likely this amino acid has a lower role during the peptide -DNA complex formation and is not an active participant in the binding site. For example, comparison of the ATHP3 and the R2A/R4A/R8A/W10A peptides shows higher similarities and correlation values, which suggest that these amino acids are less likely to regulate and participate in the peptide-DNA complex interaction. Moreover, P3A/R6A/P7A showed larger differences and lower correlation values, which suggest that there are most likely involved in the peptide-DNA complex interaction. These observations are in good agreement with previous NMR results that suggest that the -PRGRP- core most likely interacts with the DNA^22^. A crystal structure of the ATHP3 in complex with duplex B-form DNA revealed that the central core arginine residues protrude inward creating hydrogen bonds from the amino acid NH group to the thymine oxygen atom^23^. Nevertheless, it should be pointed out that multiple conformations can lead to a similar CCS (or IMS bands) and in the case of the peptide-DNA complexes, the largest contribution to the total CCS is from the DNA.

**Figure 3:**
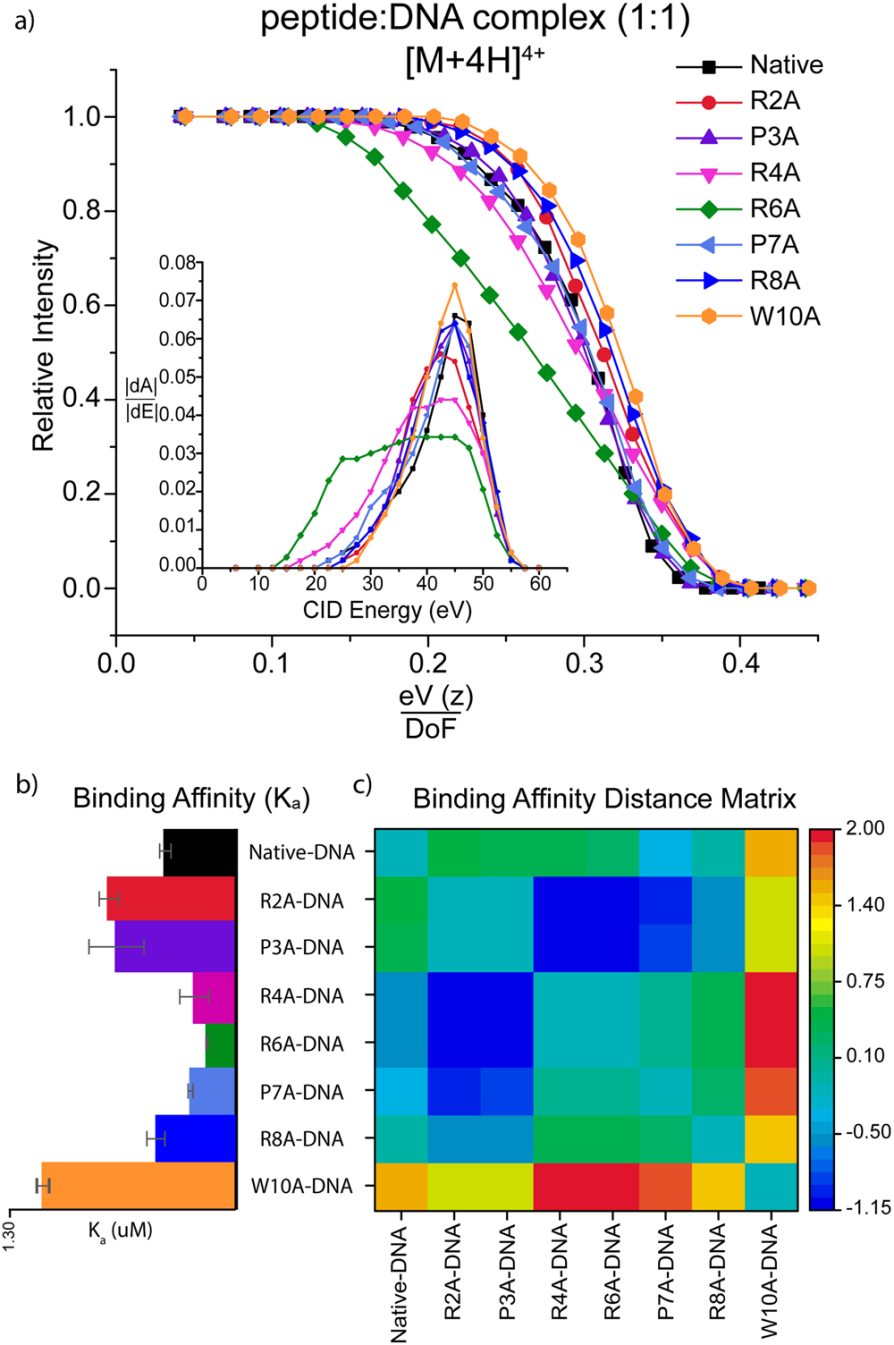
Typical CID profiles (**a**) showing the decrease in intensity of the [M + 4 H]^4+^ peptide**:** DNA complexes as a function of collision energy (eV), charge (*z*) and degrees of freedom (DoF). The dissociation threshold (inset) shows the absolute derivative of each CID profiles as a function of the collision energy. The binding affinity (**b**) and distance matrix (**c**) illustrates the influence of the single amino acid substitution on the peptide-DNA binding affinity.

A complementary way to evaluate the influence of each amino acid on the ATHP3: DNA complex is through the inspection of the dissociation curve as a function of the collision energy (Fig. 3a). The assumption is that the rupture of the intramolecular interactions that stabilizes the peptide-DNA complex using single amino acid substitutions will result in different collision energies required to disrupt the complex. Inspection of Fig. 3a shows that the R6A peptide presents the largest change in dissociation profiles, which suggest that the arginine in the sixth position is essential for the stabilization of the ATHP3: DNA complex in the -RGR- core, in good agreement with previous NMR observations^22,47^.

The probability of peptide: DNA complex formation (or binding affinity) can also be evaluated by the differences in the relative abundance of the product (i.e., complex) with respect to the initial reactants (i.e., peptide and DNA) using equations 4 and 5 (Fig. 3b,c). Inspection of Fig. 3b,c shows that the substitutions R2A/P3A/W10A has higher binding affinity (K_a_) relative to the ATHP3 peptide, while the substitutions R4A/R6A/P7A has lower binding affinity compared to the ATHP3. These results suggest that R4A/R6A/P7A are directly related to the ATHP3: DNA complex formation.

## Conclusion

The intrinsically disordered ATHP3 peptide in complex with DNA was studied using single amino acid substitutions and trapped IMS-MS at native conditions. Seven amino acid substitutions were considered and changes in the peptide and peptide-DNA complexes conformational space as well as their relative abundance and dissociation kinetics were used to establish the amino acid positions that stabilize the ATHP3:DNA complex formation in solution. The high resolution of the trapped IMS (TIMS) analyzer permitted the observation of multiple IMS bands for the intrinsically disordered ATHP3 peptide (at least 10 conformers). Using the corrected IMS profiles, the proline orientations (*cis-trans*), protonation site (i.e., basic residues and N-terminal), and side chain orientation (W10) per IMS band of the native ATHP3 were determined, providing a detailed description of the intramolecular interactions that drive the native ATHP3 conformational space. That is, these studies provided significant insight on the critical role of *cis* and *trans* proline configurations, charge location, and W10 coordination in the populations of ATHP3 structures that are observed in native conditions.

The study of peptide-DNA complexes suggests that the -RGRP- core is essential for stabilizing ATHP3: DNA complexes at native conditions. Moreover, results showed that the weakest peptide: DNA complex is formed with R6A, suggesting that this basic residue plays the most important role during binding to AT-rich regions of the DNA, in good agreement with previous NMR and X-ray data^22,23^.

It should be noted that the trapped IMS-MS workflow permits the study of multiple kinetically trapped conformations simultaneously, in contrast to the most abundant (or energetically favored) typically described using NMR and X-Ray measurements. This makes trapped IMS-MS a powerful platform for the study of intrinsically disordered biomolecules and their kinetically trapped conformers. This work highlights the potential of trapped IMS-MS combined with single amino acid substitutions for the evaluation of the peptide sequence influence on the secondary structure of peptides at native conditions. When combined with CID-MS, this workflow can be translated to the study of peptide/protein -ligand complexes. A potential limitation of the workflow is the need for higher mobility resolution as the size and complexity of the biological system increases. With the high resolving power of the TIMS analyzer (R up to 400), we estimate that this workflow can be easily extended to peptides with 35–40 amino acid residues.

## Electronic supplementary material

Supplementary Information
