# Supplementary material for: Peptide Sequence Influence on the Conformational Dynamics and DNA binding of the Intrinsically Disordered AT-Hook 3 Peptide
Source: Sci Rep. 2018 Jul 17;8:10783. doi: 10.1038/s41598-018-28956-z (PMC6050235; doi:10.1038/s41598-018-28956-z)
Supplement: Supplementary file 1 — Supplementary Information [file 41598_2018_28956_MOESM1_ESM.docx]

Supporting Information

**Peptide Sequence Influence on the Conformational Dynamics and DNA binding of the Intrinsically Disordered AT-Hook 3 Peptide**

Alyssa Garabedian^†^, Alexander Bolufer^†^, Fenfei Leng^†,‡^ and Francisco Fernandez-Lima^†,‡^*.

*†Department of Chemistry and Biochemistry, Florida International University, Miami, Florida 33199, United States*

*‡Biomolecular Sciences Institute, Florida International University, Miami, Florida 33199, United States*

Corresponding Author

[fernandf@fiu.edu](mailto:fernandf@fiu.edu)

**Table of contents:**

**Table S1.** ATHP3 single amino acid substitutions considered and their nomenclature.

**Figure S1.** Schematic diagram of the TIMS cell used to trap and separate ions based on differences in each species ion neutral collision cross section.

**Figure S2.** Typical, normalized ATHP3 and variant mobility profiles for the [M+H]+, [M+2H]2+ and [M+3H]3+(a). Peptide IMS fingerprint (b) and correlation matrix (c) are used for assessment of the primary sequence effect on the secondary structure

**Figure S3.** Typical ion mobility profiles (a) of the native and substituted ATHP3 : DNA complexes. The IMS profile fingerprint (b) of the complexes was used to generate a correlation matrix (c).

**Table S1.** ATHP3 single amino acid substitutions considered and their nomenclature.

| Peptides | Peptide sequence and position of amino acids | | | | | | | | | |
| --- | --- | --- | --- | --- | --- | --- | --- | --- | --- | --- |
|  | 1 | 2 | 3 | 4 | 5 | 6 | 7 | 8 | 9 | 10 |
| Native | Lys (K) | Arg (R) | Pro (P) | Arg (R) | Gly (G) | Arg (R) | Pro (P) | Arg (R) | Lys (K) | Trp (W) |
| R2A | Lys | Ala (A) | Pro | Arg | Gly | Arg | Pro | Arg | Lys | Trp |
| P3A | Lys | Arg | Ala | Arg | Gly | Arg | Pro | Arg | Lys | Trp |
| R4A | Lys | Arg | Pro | Ala | Gly | Arg | Pro | Arg | Lys | Trp |
| R6A | Lys | Arg | Pro | Arg | Gly | Ala | Pro | Arg | Lys | Trp |
| P7A | Lys | Arg | Pro | Arg | Gly | Arg | Ala | Arg | Lys | Trp |
| R8A | Lys | Arg | Pro | Arg | Gly | Arg | Pro | Ala | Lys | Trp |
| W10A | Lys | Arg | Pro | Arg | Gly | Arg | Pro | Arg | Lys | Ala |


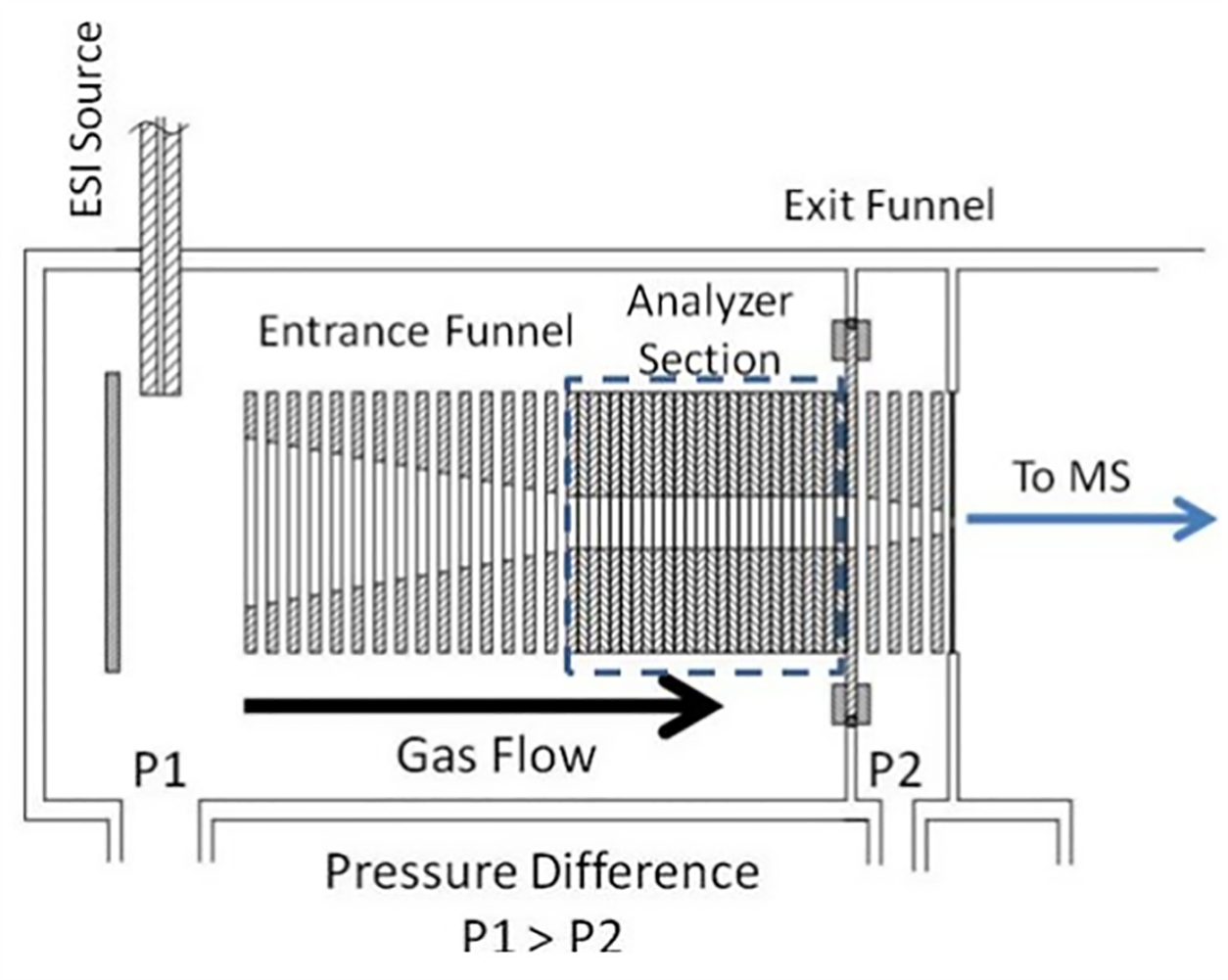


**Figure S1.** Schematic diagram of the TIMS cell used to trap and separate ions based on differences in each species ion neutral collision cross section.


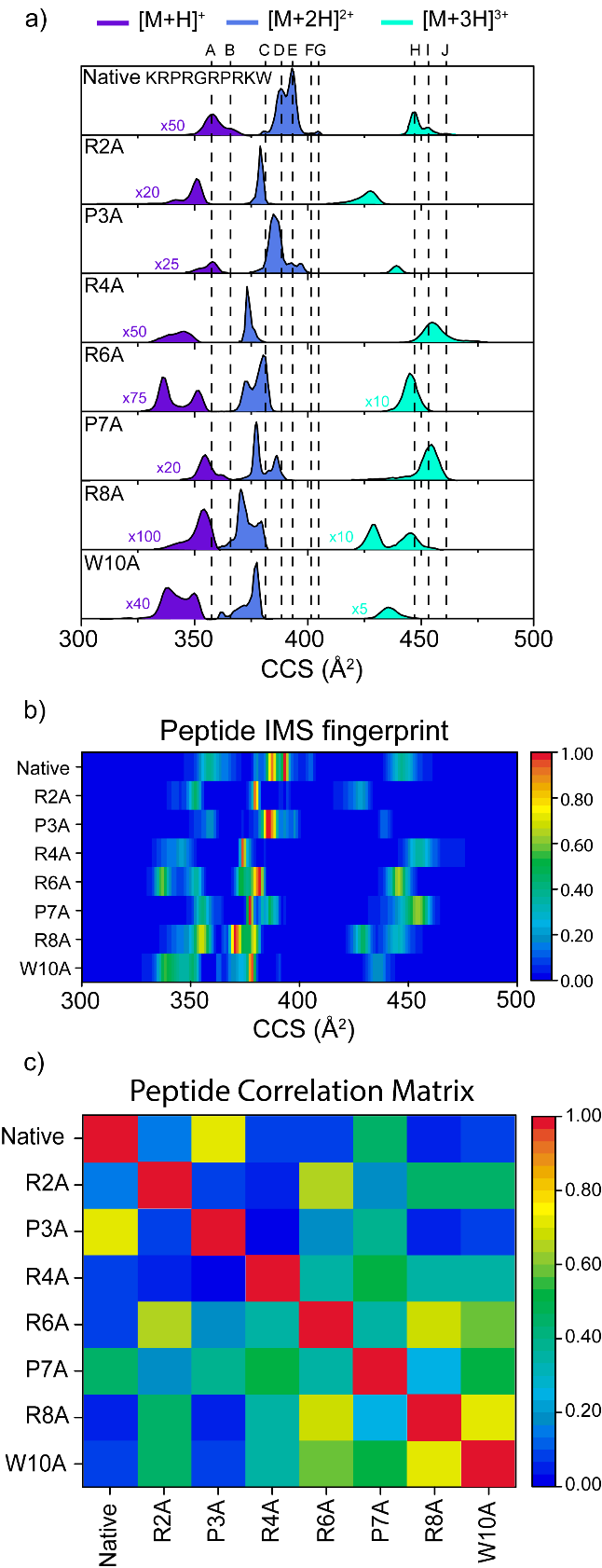


**Figure S2.** Typical, normalized ATHP3 and variant mobility profiles for the [M+H]+, [M+2H]2+ and [M+3H]3+(a). Peptide IMS fingerprint (b) and correlation matrix (c) are used for assessment of the primary sequence effect on the secondary structure


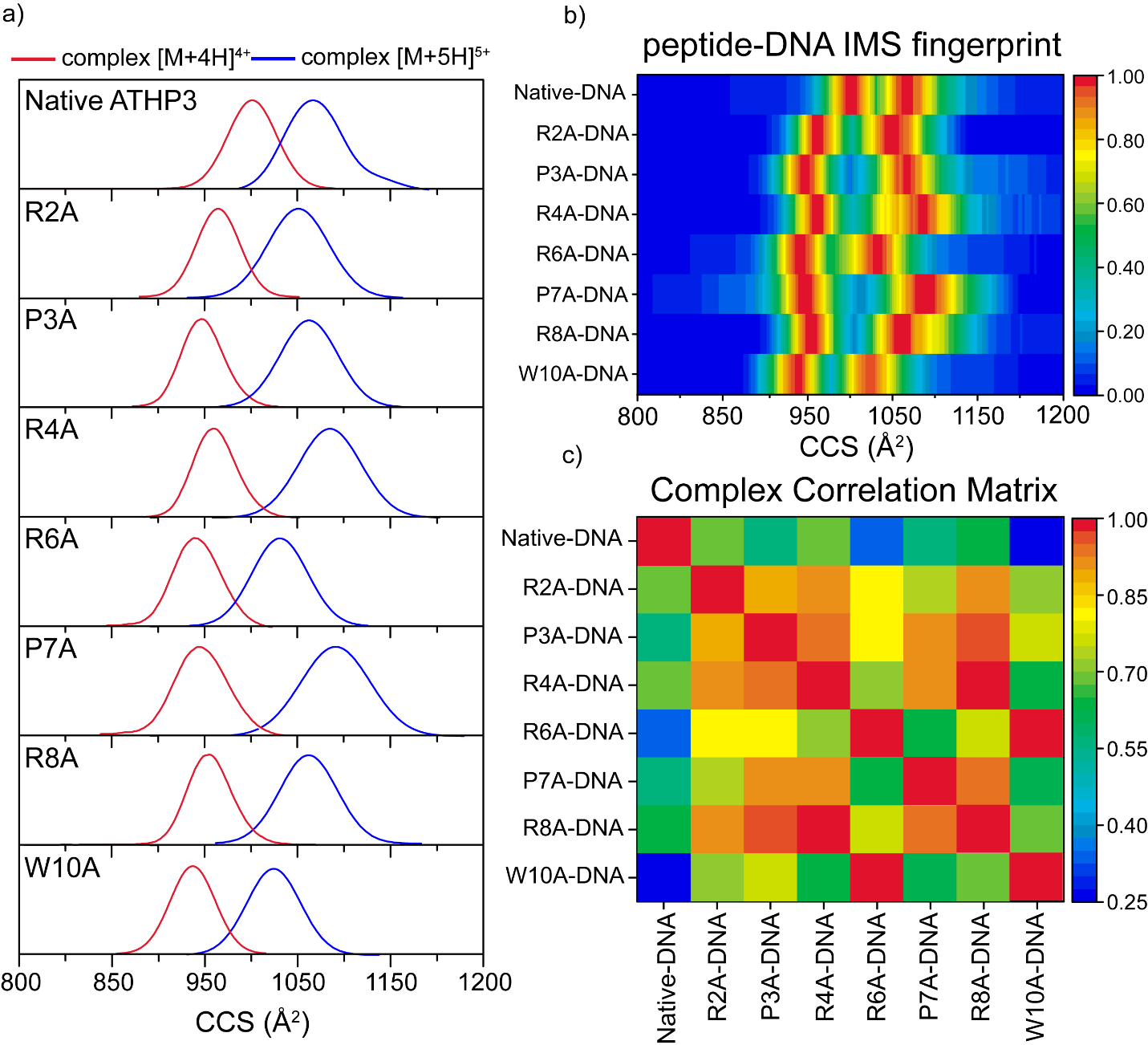


**Figure S3.** Typical ion mobility profiles (a) of the native and substituted ATHP3 : DNA complexes. The IMS profile fingerprint (b) of the complexes was used to generate a correlation matrix (c).

**Experimental Methods**

*Sample preparation.* Native AT-hook peptide 3 (Lys-Arg-Pro-Arg-Gly-Arg-Pro-Arg-Lys-Trp) and all amino acid substituted peptides were purchased from GenScript and used without further purification. An AT-rich DNA oligomer, denoted as FL876, sequence GGATATTGCCCCCGCAATATCC (C_212_H_270_N_79_O_130_P_21_, MW 6655.1561) was purchased from Eurofins Genomics (Luxembourg City, Luxembourg) and used as received. This 22 nucleotide DNA hairpin contains a 9 base pair stem comprised of a 5 base pair AT rich region in the middle of the stem. Solvents and ammonium acetate salts utilized in this study were analytical grade or better and purchased from Fisher Scientific (Pittsburgh, PA). A Tuning Mix calibration standard (G24221A) was obtained from Agilent Technologies (Santa Clara, CA) and used as received.

*Peptide nomenclature.* The amino acid sequence of the ATHP3 and variants are presented in Table S1, along with the nomenclature followed throughout the text. Specifically, the variant peptides are referred to by the original amino acid residue followed by their position and the replacement amino acid in a single-code nomenclature.

*Ion Mobility Spectrometry-Mass Spectrometry.* Details regarding the trapped IMS (TIMS) operation and specifics compared to traditional IMS can be found elsewhere.*^33, 36-39^* Briefly, a custom nESI-TIMS unit was coupled to a Maxis Impact Q-TOF mass spectrometer (Bruker, Billerica, MA). The TIMS unit is run by custom software in LabView (National Instruments) synchronized with the MS platform controls. Sample aliquots (10 μL) were loaded in a pulled-tip capillary biased at 700-1200 V to the MS inlet. The nitrogen bath gas flow is defined by the pressure differential between the entrance funnel (*P_1_* = 2.6 mbar) and the exit funnel (*P_2_* = 1.1 mbar) at *ca.* 294 K. A 880 kHz and 200 V_pp_ RF potential was applied. Deflector, capillary, entrance funnel, entrance and exit analyzer voltages were 60, 50, 0, -200-0, and 60 V in positive mode (and -60, -50, 200-0, and -60 V in negative mode) to prevent ion heating prior to IMS separation. The reduced mobility, K, of an ion in a TIMS cell is described by:

$K= \frac{V_{g}}{E}\frac{A}{(V_{elution}-V_{out})}$ (1)

where *v_g_*, *E*, *V_elution_* and *V_out_* are the gas velocity, applied electric field, elution voltage and exit analyzer voltage, respectively. After thermalization, species were eluted from the TIMS cell by decreasing the electric field in stepwise decrements (referred to as the “ramp”) and can be described by a characteristic elution voltage (*V_elution_*). The mobility calibration constant *A* was determined using known reduced mobilities of Tuning Mix components (*K_0_* of 1.013, 0.835, and 0.740 cm^2^/(V.s) for respective *m/z* 622, 922, and 1222). The scan rate (*Sr*=Δ*V_ramp_*/*t_ramp_*) was optimized for every experiment.

The measured mobilities were converted into CCS (Ω, Å²) using the Mason-Schamp equation:

$\Omega=\frac{{(18\pi)}^{1/2}}{16}\frac{z}{(k_{B}{T)}^{1/2}}\left[ \frac{1}{m_{i}} \right.+\left. \frac{1}{m_{b}} \right]^{1/2}\frac{1}{K_{0}}\frac{1}{N^{*}}$ (2)

where z is the charge of the ion, k_B_ is the Boltzmann constant, N^*^ is the number density of the bath gas and *m_i_* and *m_b_* refer to the masses of the ion and bath gas, respectively. TIMS-MS spectra were analyzed using Compass Data Analysis 5.0 (Bruker Daltonik GmbH) and TIMS Data Viewer 1.4.0.31397 (Bruker Daltonik GmbH). The IMS corrected profiles were compared using the correlation coefficient function:

$Correl (X, Y)=\frac{\sum\left( x-\bar{x} \right)(y-\bar{y})}{\sqrt{{\sum{(x-\bar{x})}^{2}(y-\bar{y})}^{2}}}$ (3)

where $\bar{x}$ and $\bar{y}$ are the sample means average for IMS profile 1 and 2.

*Correction of amino acid substituted* *collision cross sections.* For direct comparison of the mobility profiles between the ATHP 3 and single amino acid substituted peptides (e.g. Arg to Ala, Pro to Ala and Trp to Ala), the CCS profiles were adjusted based on the method previously described in references.^26, 40, 41^ To account for differences in CCS between N_2_ and He, the CCS_N2_ = 1.0857 (CCS_He_) + 81.459 [Å^2^] conversion was used.^42, 43^ This resulted in Arg to Ala, Pro to Ala and Trp to Ala substitutions to be corrected by 17.43 Å^2^, 2.68 Å^2^ and 17.43 Å^2^ in N_2_, respectively. Because the tryptophan residue CCS has not been previously reported, the Arg value was used instead considering the similarity between the geometries and steric hindrances.

*Determination of binding affinities.* Peptide-DNA binding affinities (K_a_) were calculated, using the general equation for an association reaction, based on the peak area of the complex divided by the peak area of the unbound DNA and unbound peptide.^44^

K_a_ = $\frac{(Complex)}{(DNA)(Peptide)}$ (4)

A distance matrix was utilized to better evaluate relative changes associated with the single amino acid substitution using the equation:

D(X,Y)=$\left[ \frac{K_{a\left( {peptide}_{X} \right)}-K_{a({peptide}_{Y})}}{< K_{a_{peptide (1-8)}}>} \right]$ (5)
